# Supplementary material for: Gut microbiome signatures of vegan, vegetarian and omnivore diets and associated health outcomes across 21,561 individuals
Source: Nat Microbiol. 2025 Jan 6;10(1):41–52. doi: 10.1038/s41564-024-01870-z (PMC11726441; doi:10.1038/s41564-024-01870-z)
Supplement: Supplementary file 1 — Reporting Summary [file 41564_2024_1870_MOESM1_ESM.pdf]

nature portfolio

Corresponding author(s): Nicola Segata

Last updated by author(s): Oct 9, 2024

Reporting Summary

Nature Portfolio wishes to improve the reproducibility of the work that we publish. This form provides structure for consistency and transparency in reporting. For further information on Nature Portfolio policies, see our Editorial Policies and the Editorial Policy Checklist.

Statistics

For all statistical analyses, confirm that the following items are present in the figure legend, table legend, main text, or Methods section.

n/a	Confirmed	
☐	☒	The exact sample size (*n*) for each experimental group/condition, given as a discrete number and unit of measurement
☐	☒	A statement on whether measurements were taken from distinct samples or whether the same sample was measured repeatedly
☐	☒	The statistical test(s) used AND whether they are one- or two-sided<br>*Only common tests should be described solely by name; describe more complex techniques in the Methods section.*
☐	☒	A description of all covariates tested
☐	☒	A description of any assumptions or corrections, such as tests of normality and adjustment for multiple comparisons
☐	☒	A full description of the statistical parameters including central tendency (e.g. means) or other basic estimates (e.g. regression coefficient) AND variation (e.g. standard deviation) or associated estimates of uncertainty (e.g. confidence intervals)
☐	☒	For null hypothesis testing, the test statistic (e.g. *F*, *t*, *r*) with confidence intervals, effect sizes, degrees of freedom and *P* value noted<br>*Give P values as exact values whenever suitable.*
☒	☐	For Bayesian analysis, information on the choice of priors and Markov chain Monte Carlo settings
☒	☐	For hierarchical and complex designs, identification of the appropriate level for tests and full reporting of outcomes
☐	☒	Estimates of effect sizes (e.g. Cohen's *d*, Pearson's *r*), indicating how they were calculated

Our web collection on statistics for biologists contains articles on many of the points above.

Software and code

Policy information about availability of computer code

Data collection	n/a
Data analysis	R (version 4.2.2), MetaPhlAn 4 (version 4.beta.2, database vJan21_CHOCOPhlAnSGB_202103), HUMAnN (version 3.6), scikit-learn python library (version 0.22.2). The code for the analyses conducted here is provided in Supplementary File 1. The pooled estimate of effect sizes from linear models was computed based on the pipeline https://github.com/waldronlab/curatedMetagenomicDataAnalyses/blob/main/python_tools/metaanalyze.py. An importable meta-analysis python library is also freely available at https://github.com/SegataLab/inverse_var_weight/blob/main/meta_analyses.py.

For manuscripts utilizing custom algorithms or software that are central to the research but not yet described in published literature, software must be made available to editors and reviewers. We strongly encourage code deposition in a community repository (e.g. GitHub). See the Nature Portfolio guidelines for submitting code & software for further information.

Data

Policy information about availability of data

All manuscripts must include a data availability statement. This statement should provide the following information, where applicable:

- Accession codes, unique identifiers, or web links for publicly available datasets
- A description of any restrictions on data availability
- For clinical datasets or third party data, please ensure that the statement adheres to our policy

The publicly available datasets used in this work are available from their respective publications in Tarallo et al. 2022 and De Filippis et al. 2019. Raw metagenomic samples are provided for all participants of the ZOE PREDICT Studies. Specifically, PREDICT 1 has already been made publicly available as reported previously (Asnicar, Berry, et al. 2021) under the NCBI-SRA bioproject ID PRJEB39223, whereas the PREDICT 2 is deposited in EBI under accession number PRJEB75460, and PREDICT 3 cohorts under EBI accession numbers PRJEB75463 and PRJEB75464. Sex, age, BMI, country, and the quantitative taxonomic profiles are available for each sample within the curatedMetagenomicData package Pasolli et al. 2017. The ZOE Microbiome Rankings for the full list of species are made available (and kept up-to-date) at https://zoe.com/our-science/microbiome-ranking and in their current version are reported in Supplementary Table 14. ZOE is the owner of the pseudonymized data and metadata and researchers interested in follow-up studies requiring additional specific metadata information should fill out a research request proposal at https://zoe.com/our-science/collaborate [to appear upon acceptance] that will be evaluated by a sub-panel of the ZOE Scientific Advisory Board once per month for their priority, relevance and in compliance with privacy and data protection regulations.

Research involving human participants, their data, or biological material

Policy information about studies with human participants or human data. See also policy information about sex, gender (identity/presentation), and sexual orientation and race, ethnicity and racism.

Reporting on sex and gender	Data on sex (not gender) were collected with informed consent of participants (see Data Availability Statement). Sex was considered throughout the analysis as an explanatory variable, as is indicated throughout the Methods.
Reporting on race, ethnicity, or other socially relevant groupings	Data on socially relevant variables such as race or ethnicity were neither collected nor considered in this research, where the focus was on dietary patterns pertaining to veganism, vegetarianism and mixed diets.
Population characteristics	Participants were aged 52 +/- 12.5 years (mean +/- standard deviation). Genotype and diagnosis information was not collected.
Recruitment	Information pertaining to the publicly available datasets used in this work are available from their respective publications: (Tarallo et al. 2022) and (De Filippis et al. 2019). Participants of P1, P2, P3 US22A, and P3 UK22A all gave informed study consent either written or electronically. In addition, P3 US22A and P3 UK22A participants gave product research consent during the course of product purchase at ZOE Ltd. Only the US subset of P1 received modest direct financial compensation for their participation. All other participants did not receive direct financial compensation beyond reimbursement of expenses incurred.
Ethics oversight	Information pertaining to the publicly available datasets used in this work are available from their respective publications: Asnicar et al. 2021, Tarallo et al. 2022 and De Filippis et al. 2019. Both P3 plus P2 clinical trials were registered at https://www.clinicaltrials.gov (clinical trial identifier for P3: NCT04735835; P2: NCT03983733) and ethical approval was obtained (P3 US protocol number (IRB): Pro00044316; P3 UK ethical review reference: HR-23/24-28300; P2 IRB: Pro00033432).

Note that full information on the approval of the study protocol must also be provided in the manuscript.

Field-specific reporting

Please select the one below that is the best fit for your research. If you are not sure, read the appropriate sections before making your selection.

☒ Life sciences ☐ Behavioural & social sciences ☐ Ecological, evolutionary & environmental sciences

For a reference copy of the document with all sections, see nature.com/documents/nr-reporting-summary-flat.pdf

Life sciences study design

All studies must disclose on these points even when the disclosure is negative.

Sample size	This study encompassed two published, publicly available datasets (Tarallo et al. 2022 with 118 individuals and De Filippis et al. 2019 with 97 individuals) along with three ZOE PREDICT datasets: P1, a UK cohort with 1,062 individuals; P3 UK22A a UK cohort with 12,353 individuals; and P3 US22A, a US cohort with 7,931 individuals. In total, 656 vegans, 1,088 vegetarians, and 19,817 omnivores were sampled. When possible, we also included samples from the ZOE PREDICT 2 (P2) cohort, which encompassed only omnivores from the US (843 individuals), thus limiting its usability in this analysis.
Data exclusions	Data was only excluded in the alpha diversity analysis, in which we considered all observations outside the 95% CI to be outliers, which removed 22 out of the 21,561 samples.
Replication	The gut microbial signatures of the 3 diet patterns were replicable across 5 independent cohorts (P1, P3 US22A, P3 UK22A, De Filippis et al.

Replication	(2019) and Tarallo et al. (2022) using a cross-LODO machine learning approach. Linking these patterns to FFQs and individual physiological data was replicable across 4 cohorts for which FFQ data were present (P1, P2, P3 US22A, and P3 UK22A) using a meta-analytical approach.
Randomization	Participants were grouped into dietary patterns according to their reported dietary patterns. Randomization was only necessary when cross-LODO machine learning was applied, in which case a per-cohort (ten-times, ten-folds) cross-validation was performed, in which the rest of the cohorts is added to each training set as a support.
Blinding	Authors who extracted and sequenced stool samples did not conduct the microbiome analysis.

Reporting for specific materials, systems and methods

We require information from authors about some types of materials, experimental systems and methods used in many studies. Here, indicate whether each material, system or method listed is relevant to your study. If you are not sure if a list item applies to your research, read the appropriate section before selecting a response.

Materials & experimental systems

n/a	Involved in the study
☒	☐ Antibodies
☒	☐ Eukaryotic cell lines
☒	☐ Palaeontology and archaeology
☒	☐ Animals and other organisms
☒	☐ Clinical data
☒	☐ Dual use research of concern
☒	☐ Plants

Methods

n/a	Involved in the study
☒	☐ ChIP-seq
☒	☐ Flow cytometry
☒	☐ MRI-based neuroimaging

Plants

Seed stocks	n/a
Novel plant genotypes	n/a
Authentication	n/a
